# Clinical Characteristics and Optimal Therapy of Acute Myeloid Leukemia with Myelodysplasia-Related Changes: A Retrospective Analysis of a Cohort of Chinese Patients

**DOI:** 10.4274/tjh.galenos.2021.2021.0009

**Published:** 2021-08-25

**Authors:** Lei Wang, Xiaoxia Chu, Jingyao Wang, Licai An, Yinghui Liu, Li Li, Junqing Xu

**Affiliations:** 1 Qingdao University Medical College, Affiliated Yantai Yuhuangding Hospital, Department of Hematology, Yantai, China; 2 Linyi Central Hospital, Department of Hematology, Linyi, China

**Keywords:** Acute myeloid leukemia with myelodysplasia-related changes, Clinical characteristics, Therapy

## Abstract

**Objective:**

This study aimed to investigate the clinical characteristics of acute myeloid leukemia with myelodysplasia-related changes (AML-MRC) according to the 2016 World Health Organization classification and the preferred therapy for patients with AML-MRC aged 60-75 years.

**Materials and Methods:**

We retrospectively analyzed differences in clinical data among 190 patients with AML-MRC and 667 patients with AML not otherwise specified (AML-NOS). We also compared different therapeutic regimens among patients with AML-MRC aged 60-75 years.

**Results:**

Compared with AML-NOS, patients with AML-MRC had significantly different clinical characteristics as well as worse overall survival (OS) (9.2 vs. 13.6 months; p<0.001) and complete remission rates (65.3% vs. 76.2%; p=0.005). Multivariate analysis performed for the whole group (patients with both AML-MRC and AML-NOS) showed that AML-MRC was the independent prognostic factor (p=0.002). Additional multivariate analysis performed for 190 patients with AML-MRC indicated that age (p<0.001) and lactate dehydrogenase (p=0.031) were independent prognostic factors. Compared with the IA/DA regimen [idarubicin and cytarabine (IA) or daunorubicin and cytarabine (DA)], the DAC+CAG regimen [decitabine and half-dose CAG regimen (cytarabine, aclarubicin, and granulocyte colony-stimulating factor)] was associated with better OS (4.5 vs. 6.2 months; p=0.021) in patients aged 60-75 years and categorized into the unfavorable risk group.

**Conclusion:**

AML-MRC cases exhibited worse clinical outcomes compared to AML-NOS. Compared to the IA/DA regimen, the DAC+CAG regimen was the optimal choice for patients with AML-MRC in the unfavorable risk group and aged 60-75 years.

## Abstract (tr)

**Amaç:**

Bu çalışma, 2016 Dünya Sağlık Örgütü sınıflamasına göre myelodisplazi ilişkili değişiklikler gösteren akut myeloid löseminin (AML-MRC) klinik özelliklerini ve 60-75 yaş arası AML-MRC hastalarında tercih edilen tedaviyi araştırmayı amaçlamıştır.

**Gereç ve Yöntemler:**

AML-MRC’li 190 hasta ve başka şekilde tanımlanmamış AML’li (AML-NOS) 667 hasta arasındaki klinik farklılıkları geriye dönük olarak analiz ettik. Ayrıca 60-75 yaş arası AML-MRC’li hastalar arasında farklı terapötik rejimleri karşılaştırdık.

**Bulgular:**

AML-NOS ile karşılaştırıldığında, AML-MRC’li hastalar, daha kötü genel sağkalım (GS) (9,22’ye karşı 13,6 ay; p<0,001) ve daha düşük tam remisyon oranları ile (%65,3’e karşı %76,2; p=0,005) önemli ölçüde farklı klinik özelliklere sahipti. Tüm grup (hem AML-MRC hem de AML-NOS’li hastalar) için yapılan çok değişkenli analiz, AML-MRC’nin bağımsız prognostik faktör olduğunu gösterdi (p=0,002). AML-MRC’li 190 hasta için yapılan ek çok değişkenli analiz, yaşın (p<0,001) ve laktat dehidrogenaz düzeyinin (p=0,031) bağımsız prognostik faktör olduğunu gösterdi. IA/DA rejimi [idarubisin ve sitarabin (IA) veya daunorubisin ve sitarabin (DA)] ile karşılaştırıldığında, DAC+CAG rejimi [desitabin ve yarım doz CAG rejimi (sitarabin, aklarubisin ve granülosit koloni uyarıcı faktör)] 60-75 yaş arası ve olumsuz risk grubuna dahil olan hastalarda daha iyi GS ile ilişkili (4,5’e karşı 6,2 ay; p=0,021) bulundu.

**Sonuç:**

AML-MRC olguları, AML-NOS’ye kıyasla daha kötü klinik sonuçlar sergilemiştir. IA/DA rejimi ile karşılaştırıldığında, DAC+CAG rejimi, olumsuz risk grubundaki 60-75 yaş AML-MRC’li hastalar için optimal seçim olarak bulunmuştur.

## Introduction

Acute myeloid leukemia with myelodysplasia-related changes (AML-MRC) is a distinct entity first defined by the World Health Organization (WHO) in 2008 [1]. The 2016 WHO classification revised the myelodysplastic syndrome (MDS)-related cytogenetic abnormalities: del (9q) was removed and patients with mutated *NPM1* or biallelic *CEBPA* were recategorized as having recurrent genetic abnormalities [2]. According to recent studies, AML-MRC has a worse prognosis, including lower complete remission (CR) rate and shorter overall survival (OS), compared to AML not otherwise specified (AML-NOS) [3,4,5]. Although the IA/DA regimen [idarubicin and cytarabine (IA) or daunorubicin and cytarabine (DA)] and DAC+CAG regimen [decitabine and half-dose CAG regimen (cytarabine, aclarubicin, and granulocyte colony-stimulating factor)] have often been chosen for chemotherapy, no particular therapy has yet been found to have therapeutic advantages, especially in patients older than 60 years and not eligible for allogeneic hematopoietic stem cell transplantation (allo-HSCT). We retrospectively investigated 190 patients with AML-MRC admitted to our hospital and compared those cases with AML-NOS for a better understanding of the clinical and biological features. We also compared the IA/DA and DAC+CAG regimens in patients aged 60-75 years to determine the optimal therapy.

## Materials and Methods

### Patients

Our study was performed based on a cohort of 857 patients admitted to our hospital between August 2010 and September 2019 with complete data regarding baseline characteristics and treatment outcomes. These patients were reevaluated as having AML-NOS or AML-MRC according to the 2016 WHO classification of myeloid neoplasms and acute leukemia [2], strictly excluding cases of therapy-related myeloid neoplasms and AML with recurrent genetic abnormalities including mutated *NPM1* and biallelic *CEBPA*. Patients who underwent allo-HSCT were also excluded. Clinical and laboratory data were searched in electronic medical records. Follow-up information was obtained from electronic records or by contacting family members and was initialized from the day of diagnosis to October 1, 2020, or the day of death. All subjects provided informed consent in compliance with the Declaration of Helsinki.

### Morphology Analysis

Morphology analyses of 857 patients were confirmed by at least two morphological experts. Peripheral blood and bone marrow smears were stained using the Wright-Giemsa method. Cytochemistry was performed using myeloperoxidase, non-specific esterase, sodium fluoride inhibition tests, and periodic acid-Schiff staining. Dyserythropoiesis was confirmed when there were erythroid precursors showing megaloblastic nuclei, karyorrhexis, nuclear fragments, or multinucleation. Dysgranulopoiesis was characterized as polymorphonuclear neutrophils with agranular or hypogranular cytoplasm or with hyposegmented nuclei (pseudo-Pelger-Hüet anomaly). Dysmegakaryopoiesis was defined as micromegakaryocytes and multiple separated nuclei or monolobed nuclei in megakaryocytes of all sizes. Patients were categorized as having AML with multilineage dysplasia (AML-MLD) upon the presence of dysplasia in ≥50% cells in at least two cell lineages. All cases fulfilled the 2016 WHO criterion of at least 20% blasts in the peripheral blood or bone marrow.

### Molecular Mutation Analysis

Molecular mutation analyses including *CEBPA*, *NPM1*, *ASXL1*, *RUNX1*, and *Flt3-ITD* were obtained for the whole group. Before May 2016, this process was performed by polymerase chain reaction, which was then replaced by high-throughput sequencing.

### Cytogenetic Analysis

Cytogenetic information was obtained for all patients. Chromosome karyotype detection of bone marrow cells was performed by short-time culture and G-banding methods. Patients were categorized into an intermediate risk group or poor risk group based on cytogenetics and molecular mutation as outlined by the 2017 European Leukemia Net (ELN) criteria [6]. According to the 2016 WHO criteria [2], when ≥20% peripheral blood or bone marrow blasts are present and prior therapy has been excluded, cytogenetic abnormalities sufficient to diagnose AML-MRC are as follows: 1) complex karyotype; 2) unbalanced abnormalities of -7/del(7q), del(5q)/t(5q), i(17q)/t(17p), -13/del(13q), del(11q), del(12p)/t(12p), and idic(X)(q13); 3) balanced abnormalities of t(11;16), t(3;21), t(1;3), t(2;11), t(5;12), and t(5;7).

### Therapy

According to treatment regimens, patients with AML-MRC aged 60-75 years (n=99) were divided into three groups, including an IA/DA group (n=43), DAC+CAG group (n=49), and supportive group (n=7). Patients in the IA/DA group were treated with IA (idarubicin, 10-12 mg/m^2^, days 1-3; Ara-C, 100-200 mg/m^2^, days 1-7) or DA (daunomycin, 45-60 mg/m^2^, days 1-3; Ara-C, 100-200 mg/m^2^, days 1-7). Patients in the DAC+CAG group received decitabine (20 mg/m^2^, days 1-5), aclarubicin (10-14 mg/m^2^, days 4-7), Ara-C (10 mg/m^2^ q12h, days 4-10), and granulocyte colony-stimulating factor [5 µg/kg, days 4-10 or day 4 until white blood cell (WBC) count was more than 30x10^9^/L]. In the supportive group, patients received hydroxyurea to inhibit the proliferation of leukemia cells or only supportive care such as transfusions of blood products when necessary and anti-infection therapy when patients had symptoms of infection.

### Statistical Analysis

OS was defined as the time from diagnosis to death or the last follow-up. Comparison of quantitative data was performed using the t-test or Mann-Whitney U test. Comparison of categorical variables was performed by chi-square or Fisher’s exact tests. Kaplan-Meier methods and log-rank tests were used for survival analysis based on OS. Cox multivariate analysis was used to examine the prognostic factors of AML patients and confirm which of them was the independent factor. Values of p<0.05 were considered statistically significant. All statistical calculations were conducted with SPSS 24.0 (IBM Corp., Armonk, NY, USA).

## Results

### Risk Status of the Whole Group

Information on risk status was based on the revised 2017 ELN criteria [6]. Since patients with mutated *NPM1* or biallelic mutation of *CEBPA* were recategorized as having recurrent genetic abnormalities [2], there were no patients assigned to the favorable risk group. In the whole cohort (n=857), 689 cases (80.4%) were assigned to the intermediate risk group, accounting for the largest proportion, and the remaining 168 cases (19.6%) were classified into the unfavorable risk group. As we expected, compared with the unfavorable risk group, the intermediate risk group had better OS (13.4 vs. 6.8 months; p<0.001) and CR rates (76.5% vs. 59.5%; p<0.001).

### Subclassification of Patients with AML-MRC

Among 857 patients, 190 patients (22.2%) were diagnosed with AML-MRC according to the 2016 WHO classification [2]. There were 7 different subclassifications among AML-MRC patients. Most cases were diagnosed as AML-MRC for meeting only one criterion: 38 patients (20%) presented solely with MLD, 108 patients (56.8%) showed MDS-related cytogenetics, and 11 patients (5.8%) had prior history of MDS or myelodysplastic syndrome/myeloproliferative neoplasm (MDS/MPN). Meanwhile, 32 patients (16.8%) met two criteria: 14 (7.4%) of them had MDS-related cytogenetics and prior history of MDS or MDS/MPN, 14 (7.4%) showed MLD and MDS-related cytogenetics, and 4 (2.1%) had MLD and a history of MDS or MDS/MPN. Only one case (0.5%) had a combination of all three criteria.

### Clinical Characteristics of AML-MRC

**Figure 1:**
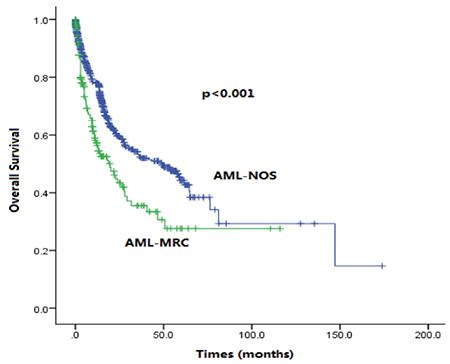
Kaplan-Meier survival rates. Overall survival for patients with acute myeloid leukemia with myelodysplasia-related changes (AML-MRC) and AML not otherwise specified (AML-NOS).

After comparing 190 patients with AML-MRC and 667 patients with AML-NOS, we found many differences between these two groups (Table 1). Patients with AML-MRC had significantly older age (p<0.001), lower hemoglobin (Hb) (p<0.001), lower WBC count (p<0.001), and higher male-to-female ratio (p=0.006) than the AML-NOS group, while no significant differences were detected in terms of platelet count (p=0.462) and lactate dehydrogenase (LDH) (p=0.139). As for clinical outcomes, compared with AML-NOS, AML-MRC patients had significantly lower CR rates (65.3% vs. 76.2%; p=0.005) and worse OS (9.2 vs. 13.6 months; p<0.001) (Figure 1). Moreover, in the intermediate risk group, the OS of AML-MRC was still worse than that of AML-NOS (9.5 vs. 13.9 months; p=0.011).

### Univariate Analysis and Multivariate Analysis

Univariate analysis was performed for the whole cohort of 857 AML patients in terms of age, WBC count, Hb, platelet count, history of MDS or MDS/MPN, MDS-related cytogenetic abnormalities, MLD, LDH, and MRC. It was found that age (p<0.001), WBC count (p<0.001), history of MDS or MDS/MPN (p=0.009), MDS-related cytogenetic abnormalities (p<0.001), MLD (p=0.002), LDH (p<0.001), and MRC (p<0.001) were prognostic factors for OS. Subsequent multivariate analysis indicated that among these factors age [hazard ratio (HR)=2.774; p<0.001], LDH (HR=1.788; p<0.001), and MRC (HR=0.653; p=0.002) were independent prognostic factors (Table 2).

In 190 patients with AML-MRC, univariate analysis suggested that LDH (p=0.031) and age (p<0.001) were prognostic factors, while WBC count, Hb, platelet count, history of MDS or MDS/MPN, MDS-related cytogenetic abnormalities, and MLD were not related to prognosis. Multivariate analysis showed that both age (HR=0.447; p<0.001) and LDH (HR=1.604; p=0.032) were independent prognostic factors for AML-MRC (Table 3).

### Treatment Analysis of Patients with AML-MRC Aged 60-75 Years

There were 99 patients aged 60-75 years with AML-MRC, who could be categorized as belonging to the intermediate risk group (n=46) or unfavorable risk group (n=53) based on the 2017 ELN criteria. We analyzed the efficacy of different treatment regimens in each group. In the intermediate risk group, no significant difference was found between the IA/DA group and DAC+CAG group with respect to CR rate (60% vs. 63.6%, p=0.808) or OS (6 vs. 6.5 months, p=0.272). However, in the unfavorable risk group, the OS of the DAC+CAG group was significantly better than that of the IA/DA group (6.2 vs. 4.5 months; p=0.021). The CR rate of the DAC+CAG group was higher than that of the IA/DA group, but the difference was not statistically significant (59.3% vs. 52.2%; p=0.406).

## Discussion

AML-MLD was first proposed in the 2001 WHO classification of myeloid neoplasms and acute leukemia and was classified as a separate category [7]. The concept was renamed as “AML-MRC” in the 2008 WHO classification [1] and myelodysplasia-related cytogenetic abnormalities as well as prior history of MDS or MDS/MPN were added as additional criteria for its recognition. Although the newly revised 2016 WHO classification has undergone some modifications, AML-MRC still includes these three categories [2], which were considered associated with poor prognosis.

We analyzed the clinical features and prognosis of 857 AML patients including 190 patients with AML-MRC and 667 patients with AML-NOS based on the 2016 WHO classification of AML [2]. Significant biological differences were found between AML-NOS and AML-MRC concerning age (p<0.001), Hb (p<0.001), and WBC count (p<0.001). Compared with AML-NOS, AML-MRC patients had significantly shorter OS (9.2 vs. 13.6 months; p<0.001) and CR rates (65.3% vs. 76.2%; p=0.005), similar to recent studies [3,4,5]. However, Devillier et al. [8] suggested that the worse prognosis of AML-MRC was probably due to unfavorable cytogenetics, which were categorized as MDS-related cytogenetics, because they assessed the prognosis of AML-NOS and AML-MRC in an intermediate risk group and found no difference between the groups for OS or relapse-free survival. On the contrary, Weinberg et al. [4] showed that AML-MRC patients had worse OS and CR rates even after excluding patients with unfavorable cytogenetics. To address this discrepancy, we carried out research similar to that of Devillier et al. [8] and obtained the opposite result: among patients of the intermediate risk group, the OS of AML-MRC patients was still significantly worse than that of AML-NOS (9.5 vs. 13.9 months; p=0.011). Moreover, our multivariate analysis showed that MRC was an independent prognostic factor after adjustment by age and MDS-related cytogenetics, coinciding with the study of Weinberg et al. [4]. The results described here support the WHO classification separating these two categories.

**Figure 2:**
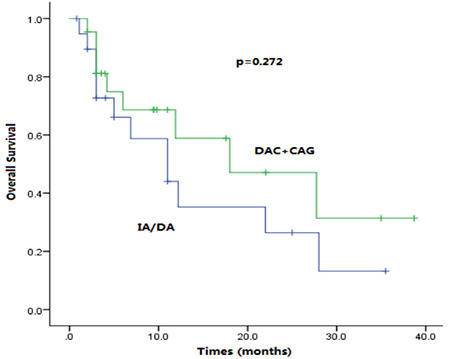
Kaplan-Meier survival rates. Overall survival for IA/DA regimen and DAC+CAG regimen in patients aged 60-75 years with acute myeloid leukemia with myelodysplasia-related changes (AML-MRC) in the intermediate risk group.

**Figure 3:**
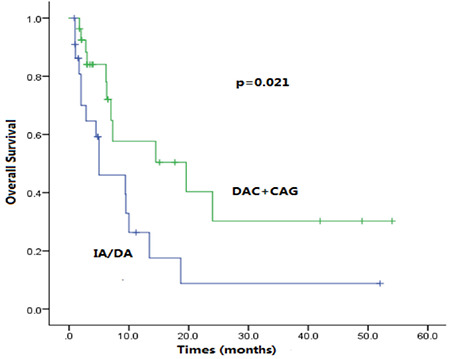
Kaplan-Meier survival rates. Overall survival for IA/DA regimen and DAC+CAG regimen in patients aged 60-75 years with acute myeloid leukemia with myelodysplasia-related changes (AML-MRC) in the unfavorable-risk group.

As described above, many well-known adverse factors were observed in cases of AML-MRC, such as older age, unfavorable cytogenetics, and multidrug-resistant phenotype, which lead to unsatisfying therapeutic response and survival. Young patients in good physical condition are offered intensive chemotherapy followed by allo-HSCT. However, the optimal chemotherapy regimen for patients older than 60 years who are not eligible for allo-HCST has always been controversial. The standard 3+7 regimen, IA or DA, is the most common induction therapy, while the CAG regimen is another common choice and is often combined with decitabine. However, comparisons of the IA/DA and DAC+CAG regimens for AML-MRC patients aged 60-75 years have rarely been reported. In our study, no significant difference was found between the two regimens (OS: 6 vs. 6.5 months, p=0.272; CR rate: 60% vs. 63.6%, p=0.808) in the intermediate risk group (Figure 2). In the poor risk group, however, the OS of patients treated with the DAC+CAG regimen was significantly longer than patients on the IA/DA regimen (6.2 vs. 4.5 months; p=0.021) (Figure 3). Therefore, we suggest that the DAC+CAG regimen should be the preferred choice for AML-MRC patients categorized into the poor risk group and aged 60-75 years.

Decitabine, a DNA-hypomethylating agent (HMA), can induce differentiation and apoptosis of leukemic blasts and activate the silenced *tumor suppressor* *gene* (TSG) impaired by the disorder of DNA methylation [9,10,11]. Recent studies have proved that decitabine is well tolerated and may improve the response rate and OS in older AML patients when used as a single agent [12,13]. When combined with other chemotherapy regimens such as CAG, IA, or HAA (homoharringtonine, cytarabine, aclarubicin), decitabine could also significantly enhance the therapeutic efficacy [14,15,16]. In 2016, Welch et al. [17] enrolled 84 patients with AML or MDS in a single-institution trial of decitabine. The results showed that the response to DAC was better among patients in the unfavorable risk group than patients in the intermediate risk/favorable risk cytogenetic group (67% vs. 34%, p<0.001), although there was no statistical difference in OS between these two groups (11.6 vs. 10 months, p=0.29). Similar findings were also reported by other researchers [18,19,20]. These studies offer a possible explanation for our result whereby patients in the unfavorable risk group benefited most from the DAC+CAG regimen. Further research will be required to determine the core mechanism of the better efficacy of DAC among patients in the unfavorable risk group.

Despite the efficacy of conventional chemotherapy, the survival of AML patients remains unsatisfactory. Over the decades, efforts made by researchers led to only minor improvements in the outcome of AML patients until the presence of several new therapies offered something fresh in the landscape of AML therapy. CPX-351, a liposomal formulation of cytarabine and daunorubicin, was approved by the US Food and Drug Administration in 2017 for the treatment of newly diagnosed therapy-related AML (tAML) or AML-MRC based on a phase III clinical trial named CLTR0310-301 [21]. In this clinical trial, CPX-351 showed better OS (9.56 vs. 5.85 months, p=0.003), better event-free survival (2.53 vs. 1.31 months, p=0.021), and higher CR rate (37.3% vs. 25.6%, p=0.016) compared to the standard 3+7 regimen in patients 60-75 years of age with newly diagnosed tAML or AML-MRC and it was found to be safe and well-tolerated [22,23,24]. Venetoclax, a selective inhibitor of the antiapoptotic protein B-cell lymphoma 2 (BCL-2), can lead to rapid initiation of apoptosis in leukemia cells [25]. When combined with low-dose cytarabine (LDAC) or HMA, venetoclax demonstrated significant improvement of CR rate and OS compared with single-agent LDAC or HMA treatment in AML patients ineligible for intensive chemotherapy, as proven by several multicenter clinical trials [26,27,28]. Based on these results, current guidelines recommend this combination as standard therapy for older and unfit patients [29]. In addition to more research on new therapies, future efforts should also be focused on reducing therapeutic toxicities for wider utilization and improving the OS of AML patients through different therapeutic combinations.

### Study Limitations

There are several limitations of our study. All data were collected in a retrospective manner and the scale of the cohort was small while analyzing the optimal choice for patients with AML-MRC aged 60-75 years in the unfavorable risk group.

## Conclusion

AML-MRC is associated with worse prognosis compared to AML-NOS and shows an independent prognostic effect. The DAC+CAG regimen may be preferred for patients aged 60-75 years who are classified in the unfavorable risk group.

## Figures and Tables

**Table 1:**
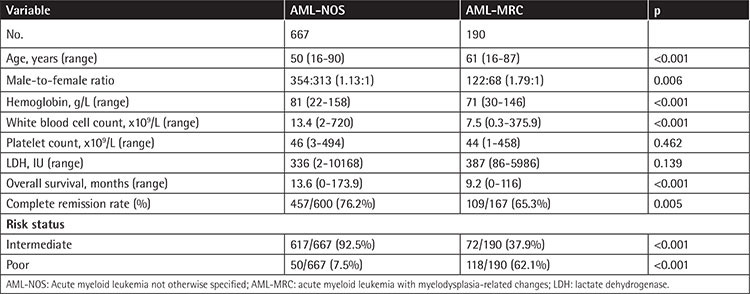
Information of the whole cohort.

**Table 2:**
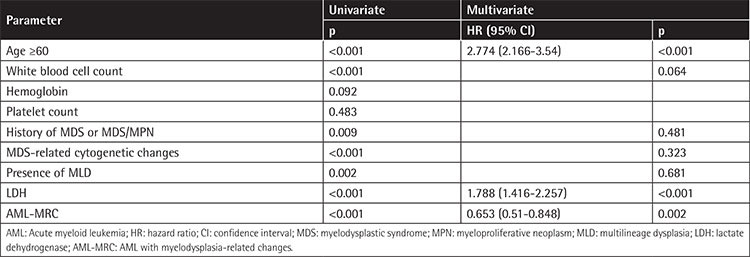
Univariate and multivariate analysis for overall survival of 864 AML patients.

**Table 3:**
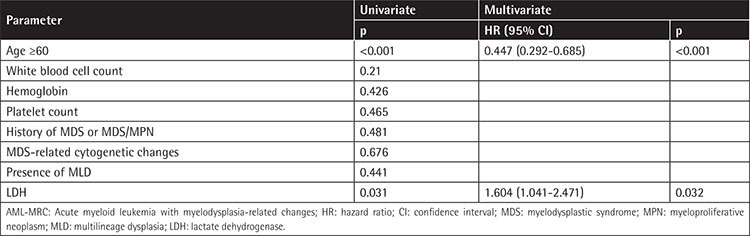
Univariate and multivariate analysis for overall survival of 191 patients with AML-MRC.
